# Supplementary material for: Identification of photoperiod-induced specific miRNAs in the adrenal glands of Sunite sheep (Ovis aries)
Source: Front Vet Sci. 2022 Jul 22;9:888207. doi: 10.3389/fvets.2022.888207 (PMC9354845; doi:10.3389/fvets.2022.888207)
Supplement: Supplementary file 1 [file Data_Sheet_1.ZIP › Supplementary file/miRDeep2 file.zip!/miRDeep2 file/Novel_Structure/LP42A2/Novel_430.pdf]

Provisional ID	:	NC_019479.2_32803
Score total	:	1.8
Score for star read(s)	:	-1.3
Score for read counts	:	0
Score for mfe	:	1.5
Score for randfold	:	1.6
Score for cons. seed	:	
Total read count	:	174
Mature read count	:	174
Loop read count	:	0
Star read count	:	0

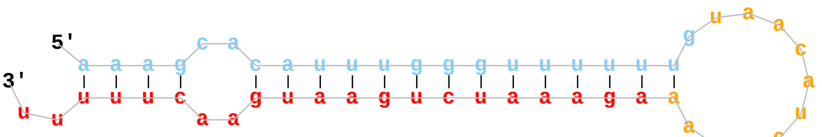

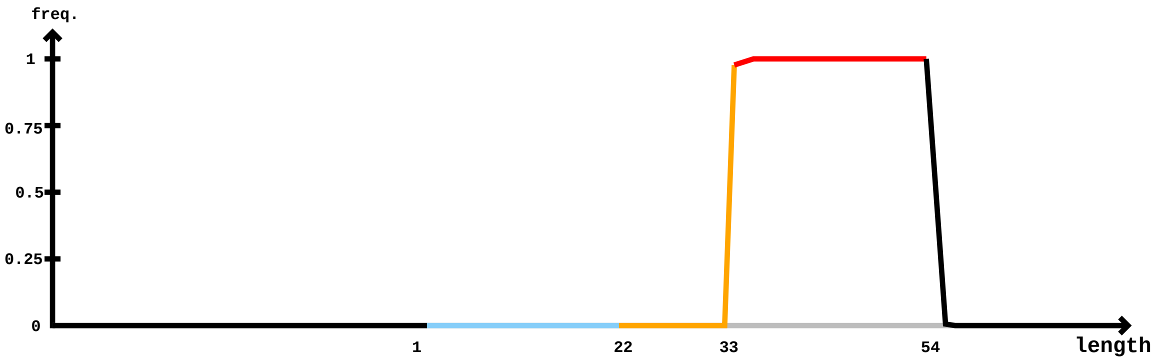


```
                                           Star                          Mature
5'- accaaaauuacagaggcuaauuuauugguuggcuaaaaagcacauuuggguuuuuuguaacaucuuaaagaaaucugaaugaacuuuuugggcaacccaauacauucua   -3'   exp
    .....................((((((((((.((((((((..((((((((((((((...........))))))))))))))..)))))))).)))))))))).......    reads   mm   sample
    ....................................................................aAaaaucugaaugaacuuuuu....................    111     1    seq
    ....................................................................aAaaaucugaaugaacuuuuug...................    59      1    seq
    .....................................................................gaaaCcugaaugaacuuuuu....................    1       1    seq
    .....................................................................gaaaucugaaugaacuuuuu....................    1       0    seq
    ......................................................................aaaucugaaugaacuuuuu....................    1       0    seq
    ......................................................................aaaucugaaCgaacuuuuugg..................    1       1    seq
```
